# *trans*-Carbonyl­chlorido­bis­(tri­ethyl­phosphane-κ*P*)platinum(II) tetra­fluorido­borate

**DOI:** 10.1107/S2414314622006071

**Published:** 2022-06-10

**Authors:** Peter W. R. Corfield

**Affiliations:** a Department of Chemistry, Fordham University, 441 East Fordham Road, Bronx, NY 10458, USA; Vienna University of Technology, Austria

**Keywords:** crystal structure, platinum, coordination compound, carbon­yl, unexpected product

## Abstract

The chemical formulation of the title compound was established as *trans*-[PtCl{P(C_2_H_5_)_3_(CO)}BF_4_ by single-crystal X-ray analysis, in contrast to the five-coordinate tetra­fluoro­ethyl­ene complex that had been anti­cipated. The compound had been prepared by reaction of *trans*-PtHCl(P(C_2_H_5_)_3_)_2_ with C_2_F_4_ in the absence of air, and the presence of the carbonyl group was not suspected. The square-planar cations and BF_4_^−^ anions are linked by C—H⋯F and C—H⋯O inter­actions into thick wavy (010) sheets. The present crystal-structure refinement is based on the original intensity data recorded in 1967.

## Abstract 2

Crystallographic details are given of an unexpected product − a platinum(II) carbonyl complex, in which the carbonyl oxygen atom has remarkably been extracted from Pyrex glassware by tetra­fluoro­ethyl­ene. Preliminary details were reported previously.

## Graphical abstract

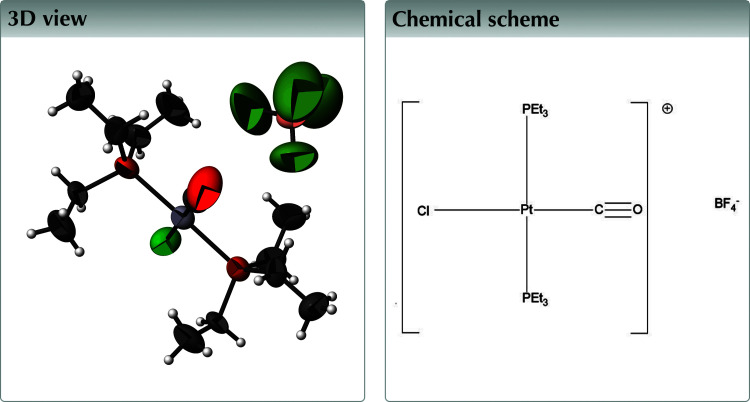

## Structure description

A low-yield product in the reaction of *trans*-PtHCl(P(C_2_H_5_)_3_)_2_ with C_2_F_4_ in the absence of air was originally postulated to be a five-coordinate platinum complex, PtHCl(π-C_2_F_4_)(P(C_2_H_5_)_3_)_2_ (Clark & Tsang, 1967 ▸), and the crystal-structure determination was undertaken at that time in view of the then current inter­est in five-coordination and of π-complexes. As described in Clark *et al.* (1967 ▸), the preliminary crystal-structure model showed no evidence of five-coordination, nor of the presence of a π-bonded tetra­fluoro­ethyl­ene group. Instead, a four-coordinated, cationic Pt^II^ complex was indicated, with a carbonyl group as the fourth ligand, isoelectronic with Vaska’s compound, IrCl(CO)(P*R*_3_)_2_ (Vaska & DiLuzio, 1961 ▸). The presence of a carbonyl group was completely unexpected, as the reaction had been carried out in a vacuum line, in the absence of oxygen. This was the first reported mol­ecular structure of a platinum carbonyl at the time, according to our database analysis below. The strong carbonyl vibrational band in the infrared spectrum was mistaken for the anti­cipated Pt—H band. Evidently, the carbonyl oxygen atom had been extracted from the Pyrex glassware by the tetra­fluoro­ethyl­ene reagent. That reaction vessels are not always as inert as they are expected to be is the subject of a recent review by Nielsen & Pedersen (2022 ▸) in which formation of the title compound in this paper is one of several examples of fluorine compounds reacting with glassware.

**Figure 1:**
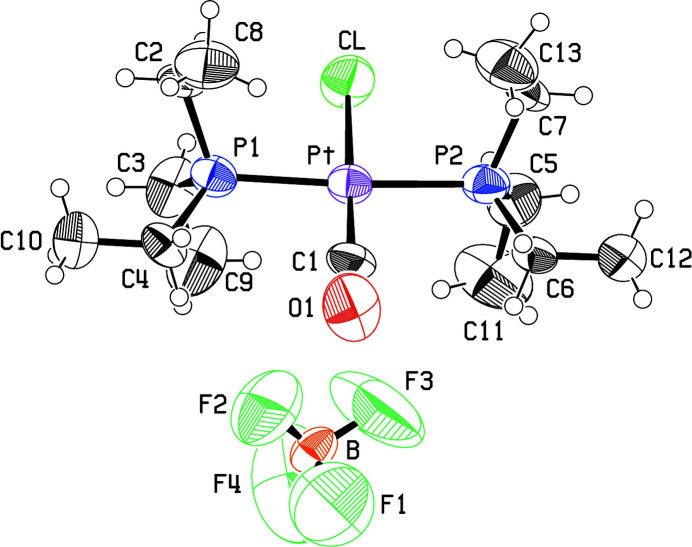
View of the mol­ecular entities showing the atomic numbering and displacement ellipsoids at the 50% probability level.

The crystal structure refinement based on the original X-ray intensity data recorded in 1967 is now presented here, because no atomic coordinates were given in the original report (Clark *et al.*, 1967 ▸) or deposited with the Cambridge Structural Database (CSD; Groom *et al.*, 2016 ▸). The square-planar platinum(II) cation and a tetra­fluorido­borate anion are shown in Fig. 1 ▸. As can be seen, the cation has an approximate mirror plane of symmetry that extends to the conformations of the ethyl groups. The Pt—CO bond length is 1.812 (17) Å, Pt—Cl is 2.301 (4) Å, and the Pt—P bond lengths are 2.341 (5) and 2.348 (5) Å. The P—Pt—CO angles average 92.9 (8)° while the Cl—Pt—P angles average 87.2 (2)°. The *trans* angles P—Pt—P and Cl—Pt—C are 174.10 (17)° and 177.0 (12)°, respectively, with the slight distortions from linearity tending towards a flattened tetra­hedron rather than a flattened square pyramid. Each of the tri­ethyl­phosphine groups has one ethyl group in the *trans* conformation and two in the *gauche* conformation*.*

**Figure 2:**
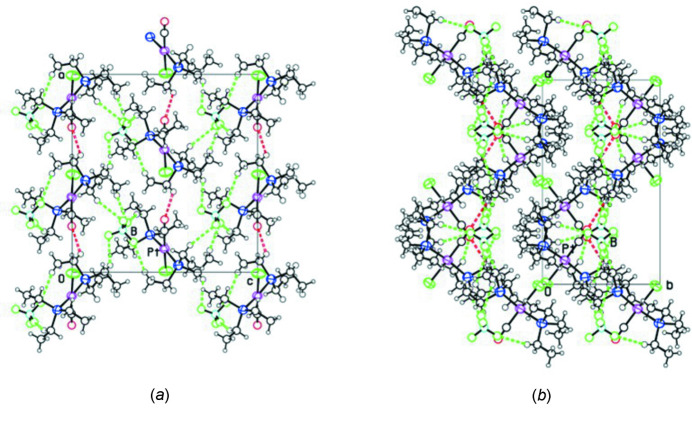
Projections of the structure down the *b* axis (*a*) and *c* axis (*b*), with arbitrary sphere sizes for the atoms. The reference cation and anion have Pt and B atoms identified. Putative C—H⋯O and C—H⋯F hydrogen bonds are shown as red and green dashed lines, respectively.

**Figure 3:**
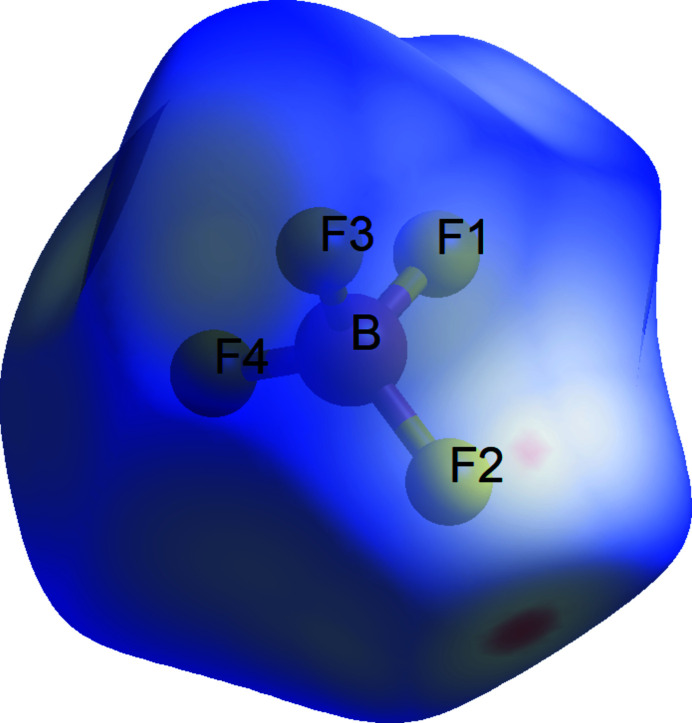
Hirshfeld *d*_norm_ surface for the BF_4_^−^ anion, showing the red area that indicates close contacts for F2.

Packing diagrams showing views down the *b* and *c* axes are shown in Fig. 2 ▸ *a* and 2*b*. There are close contacts between each tetra­fluorido­borate anion and the ethyl groups of three neighboring cations with putative C—H⋯F hydrogen bonds, as listed in Table 1 ▸. The Hirshfeld *d*_norm_ plot for the BF_4_^−^ anion shown in Fig. 3 ▸ was produced with *CrystalExplorer* (Spackman *et al.*, 2021 ▸) and indicates a close contact near F2, probably due to the C13—H13⋯F2 hydrogen bond, which seems to be the strongest C—H⋯F bond. The chlorido and carbonyl ligands do not have close inter­molecular contacts, perhaps because they are shielded by the *gauche* conformations of the neighboring ethyl groups. A putative weak C—H⋯O hydrogen bond is listed in Table 1 ▸ and shown in red in Fig. 3 ▸. The hydrogen bonds listed join cations and anions into thick wavy (010) sheets, as can be seen in Fig. 2 ▸ *b*.

**Database analysis**

From the time the preliminary structure of this compound was published in 1967, crystal and mol­ecular structures of a wide variety of platinum carbonyl complexes have been reported, ranging from metal clusters through monomeric complexes as in this case. All 662 structures found with the ‘PtCO’ search fragment in the CSD database, with all filters removed except for ‘single-crystal structure’, except the present one (TEPPTC) are dated 1968 or after. All but 20 of these structures have only one CO group coordinating to the Pt^II^ atom while the rest have just two coordinating carbonyl groups except for the [Pt(CO)_4_]^2+^ cation reported by Willner *et al.* (2001 ▸) in entry QEZTEU. The mean Pt—CO distance for the 603 structures with coordinates given is 1.860 Å, with a wide range of 1.680 to 2.095 Å. It is inter­esting that the presence of phosphine ligands tends to lead to longer Pt—CO distances, while the presence of a Cl ligand to shorter Pt—CO distances. Thus, in the 35 entries in the above structures that have two P*R*_3_ groups attached to the Pt^II^ atom as well as the CO group, the mean Pt—C distance is 1.910 Å, with a narrow range of 1.855–1.965 Å, while for the 36 entries that have a Cl as well as a carbonyl ligand, the mean Pt—CO distance is 1.837 Å with a range of 1.753 to 1.901 Å. In the latter case, the Pt—CO distance seems insensitive to whether the Cl atom is *cis* or *trans* to the CO group. These tendencies must oppose each other in the present structure, leading to the Pt—CO distance of 1.812 (17) Å. Entry GEYBOB (Rusakov *et al.*, 1988 ▸) has the same cation as in the present structure, but the anion is BF_3_Cl^−^ and there is a solvent mol­ecule in the crystal. The shape of the cation is very similar to that of the present structure, with similar distortions of the angles from 90° and a Pt—CO bond length of 1.846 Å.

## Synthesis and crystallization

A sample supplied by Dr H. C. Clark had been synthesized as described in Clark & Tsang (1967 ▸). Crystals suitable for X-ray analysis were obtained by recrystallization of the sample from methyl acetate.

## Refinement

With the early automatic diffractometer that was used to collect the original X-ray intensity data in 1967, it was not customary to obtain a set of Friedel pairs of reflections in the case of a non-centrosymmetric structure. In this case, however, due to the polar space group and the poor scattering by the small crystal, data were collected over the whole sphere of reflection up to θ = 20°; in addition, data were recollected over four quadrants for the weaker reflections at higher angles. Initial absorption corrections using a Gaussian grid were inconclusive – perhaps due to a programming error –, so for the final refinements an overall absorption correction using the tensor analysis in *XABS2* (Parkin *et al.*, 1995 ▸) was used. Hydrogen atoms were constrained, with C—H distances of 0.97 Å and 0.96 Å for CH_2_ and CH_3_ groups, respectively, and *U*_iso_(H) = 1.5*U*_eq_(C). Anisotropic temperature factors for the carbonyl CO atoms required tight restraints. While the displacement ellipsoids for the fluorine atoms are large, probably indicating some disorder for the BF_4_^−^ anion (Fig. 1 ▸), initial refinements of a disordered model were not successful and the disordered model was not pursued. There is indeed some residual electron density in the neighborhood of the BF_4_^−^ anion, but only one of the 20 highest electron density peaks in the final difference-Fourier map is near this group. Crystal data, data collection and structure refinement details are summarized in Table 2 ▸.

## Supplementary Material

Crystal structure: contains datablock(s) I. DOI: 10.1107/S2414314622006071/wm4166sup1.cif

Structure factors: contains datablock(s) I. DOI: 10.1107/S2414314622006071/wm4166Isup2.hkl

CCDC reference: 2177787

Additional supporting information:  crystallographic information; 3D view; checkCIF report

## Figures and Tables

**Table 1:** Hydrogen-bond geometry (Å, °)

*D*—H⋯*A*	*D*—H	H⋯*A*	*D*⋯*A*	*D*—H⋯*A*
C5—H5*B*⋯O1^i^	0.97	2.75	3.45 (2)	129
C13—H13*B*⋯F2^ii^	0.96	2.43	3.27 (3)	147
C4—H4*A*⋯F2	0.97	2.56	3.48 (3)	159
C7—H7*A*⋯F4^iii^	0.97	2.67	3.46 (3)	140
C11—H11*B*⋯F3	0.96	2.75	3.47 (3)	133
C6—H6*A*⋯F1^ii^	0.97	2.81	3.67 (4)	147

Symmetry codes: (i) 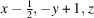 ; (ii) 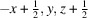 ; (iii) 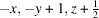 .

**Table 2:** Experimental details

Crystal data	
Chemical formula	[PtCl(C_6_H_15_P)_2_(CO)]BF_4_
*M*_r_	581.66
Crystal system, space group	Orthorhombic, *P* *c* *a*2_1_
Temperature (K)	293
*a*, *b*, *c* (Å)	16.012 (8), 9.171 (4), 14.966 (7)
*V* (Å^3^)	2197.7 (18)
*Z*	4
Radiation type	Mo *K*α
μ (mm^−1^)	6.68
Crystal size (mm)	0.12 × 0.10 × 0.08

Data collection	
Diffractometer	Picker, punched card control
Absorption correction	Empirical (using intensity measurements) (*XABS2*; Parkin *et al.*, 1995 ▸)
*T*_min_, *T*_max_	0.55, 0.81
No. of measured, independent and observed [*I* > 2σ(*I*)] reflections	7773, 3180, 2437
*R*_int_	0.062
(sin θ/λ)_max_ (Å^−1^)	0.596

Refinement	
*R*[*F* ^2^ > 2σ(*F* ^2^)], *wR*(*F* ^2^), *S*	0.041, 0.090, 0.92
No. of reflections	3180
No. of parameters	214
No. of restraints	61
H-atom treatment	H-atom parameters constrained
Δρ_max_, Δρ_min_ (e Å^−3^)	0.64, −0.76
Absolute structure	Flack *x* determined using 961 quotients [(*I* ^+^)−(*I* ^−^)]/[(*I* ^+^)+(*I* ^−^)] (Parsons *et al.*, 2013 ▸)
Absolute structure parameter	0.000 (14)

Computer programs: *PICK* (local program by J. A. Ibers), *PICKOUT* (local program by R. J. Doedens) and *EQUIV* (local program by J. A. Ibers), local version of *FORDAP*, *SHELXL* (Sheldrick, 2015 ▸), *ORTEPIII* (Burnett & Johnson, 1996 ▸; Farrugia, 2012 ▸) and *publCIF* (Westrip, 2010 ▸).

## full crystallographic data

### Crystal data

**Table:**

[PtCl(C_6_H_15_P)_2_(CO)]BF_4_	*D*_x_ = 1.758 Mg m^−^^3^*D*_m_ = 1.734 (4) Mg m^−^^3^*D*_m_ measured by flotation in CH_3_I/CCl_4_
*M**_r_* = 581.66	Mo *K*α radiation, λ = 0.7107 Å
Orthorhombic, *P**c**a*2_1_	Cell parameters from 16 reflections
*a* = 16.012 (8) Å	θ = 3.7–14.1°
*b* = 9.171 (4) Å	µ = 6.68 mm^−^^1^
*c* = 14.966 (7) Å	*T* = 293 K
*V* = 2197.7 (18) Å^3^	Needle, colorless
*Z* = 4	0.12 × 0.10 × 0.08 mm
*F*(000) = 1128	

### Data collection

**Table:**

Picker, punched card control diffractometer	*R*_int_ = 0.062
Radiation source: sealed X-ray tube	θ_max_ = 25.1°, θ_min_ = 2.2°
θ/2θ scans	*h* = 0→19
Absorption correction: empirical (using intensity measurements) (*XABS2*; Parkin *et al.*, 1995)	*k* = 0→10
*T*_min_ = 0.55, *T*_max_ = 0.81	*l* = −17→17
7773 measured reflections	3 standard reflections every 250 reflections
3180 independent reflections	intensity decay: 8(2)
2437 reflections with *I* > 2σ(*I*)	

### Refinement

**Table:**

Refinement on *F*^2^	Secondary atom site location: difference Fourier map
Least-squares matrix: full	Hydrogen site location: inferred from neighbouring sites
*R*[*F*^2^ > 2σ(*F*^2^)] = 0.041	H-atom parameters constrained
*wR*(*F*^2^) = 0.090	*w* = 1/[σ^2^(*F*_o_^2^)] where *P* = (*F*_o_^2^ + 2*F*_c_^2^)/3
*S* = 0.92	(Δ/σ)_max_ = 0.002
3180 reflections	Δρ_max_ = 0.64 e Å^−^^3^
214 parameters	Δρ_min_ = −0.76 e Å^−^^3^
61 restraints	Absolute structure: Flack x determined using 961 quotients [(*I*^+^)-(*I*^-^)]/[(*I*^+^)+(*I*^-^)] (Parsons *et al.*, 2013)
Primary atom site location: heavy-atom method	Absolute structure parameter: 0.000 (14)

### Special details

**Table:**

Geometry. All esds (except the esd in the dihedral angle between two l.s. planes) are estimated using the full covariance matrix. The cell esds are taken into account individually in the estimation of esds in distances, angles and torsion angles; correlations between esds in cell parameters are only used when they are defined by crystal symmetry. An approximate (isotropic) treatment of cell esds is used for estimating esds involving l.s. planes.

### Fractional atomic coordinates and isotropic or equivalent isotropic displacement parameters (Å^2^)

**Table:**

	*x*	*y*	*z*	*U*_iso_*/*U*_eq_	
Pt	0.11894 (3)	0.19055 (6)	0.49793 (9)	0.0540 (2)	
CL	0.0065 (3)	0.0347 (5)	0.5053 (8)	0.0887 (17)	
P1	0.1894 (3)	0.0057 (5)	0.4207 (4)	0.0580 (13)	
P2	0.0355 (3)	0.3627 (6)	0.5723 (3)	0.0606 (14)	
C1	0.2098 (10)	0.3084 (18)	0.497 (3)	0.074 (5)	
O1	0.2670 (8)	0.3789 (15)	0.498 (2)	0.114 (5)	
C2	0.1949 (12)	−0.1601 (17)	0.4862 (17)	0.068 (5)	
H2A	0.138889	−0.191260	0.502025	0.102*	
H2B	0.220505	−0.236749	0.450938	0.102*	
C3	0.1344 (12)	−0.048 (2)	0.3209 (13)	0.081 (6)	
H3A	0.167832	−0.120101	0.289379	0.122*	
H3B	0.082483	−0.095089	0.338078	0.122*	
C4	0.2941 (10)	0.045 (2)	0.3857 (11)	0.061 (5)	
H4A	0.293095	0.132343	0.349114	0.092*	
H4B	0.327255	0.066627	0.438343	0.092*	
C5	−0.0571 (12)	0.405 (2)	0.510 (2)	0.089 (7)	
H5A	−0.087502	0.315295	0.499106	0.133*	
H5B	−0.092341	0.467457	0.545982	0.133*	
C6	0.0892 (12)	0.5313 (19)	0.5972 (14)	0.071 (6)	
H6A	0.136834	0.509449	0.634974	0.107*	
H6B	0.110475	0.571661	0.541807	0.107*	
C7	−0.0026 (13)	0.295 (2)	0.6757 (12)	0.074 (6)	
H7A	−0.035167	0.370900	0.704742	0.111*	
H7B	−0.039380	0.213328	0.664405	0.111*	
C8	0.2449 (16)	−0.136 (2)	0.5696 (14)	0.100 (8)	
H8A	0.228595	−0.205293	0.614220	0.150*	
H8B	0.235030	−0.038809	0.591484	0.150*	
H8C	0.303262	−0.147316	0.556527	0.150*	
C9	0.1148 (15)	0.076 (3)	0.2576 (15)	0.117 (9)	
H9A	0.087111	0.039006	0.205509	0.175*	
H9B	0.165842	0.123467	0.240124	0.175*	
H9C	0.079213	0.145449	0.287034	0.175*	
C10	0.3376 (13)	−0.075 (2)	0.3335 (15)	0.095 (7)	
H10A	0.394947	−0.048653	0.323932	0.143*	
H10B	0.310361	−0.087509	0.276862	0.143*	
H10C	0.334969	−0.164404	0.366633	0.143*	
C11	−0.0435 (15)	0.477 (3)	0.4237 (19)	0.141 (12)	
H11A	−0.093656	0.471759	0.388736	0.212*	
H11B	0.001072	0.428995	0.392480	0.212*	
H11C	−0.028989	0.577227	0.433552	0.212*	
C12	0.0369 (16)	0.646 (2)	0.6429 (17)	0.112 (9)	
H12A	0.069086	0.733174	0.649995	0.167*	
H12B	0.019712	0.610676	0.700464	0.167*	
H12C	−0.011595	0.666085	0.607203	0.167*	
C13	0.0687 (15)	0.246 (3)	0.7400 (15)	0.100 (8)	
H13A	0.046541	0.232547	0.799019	0.150*	
H13B	0.111629	0.318759	0.741295	0.150*	
H13C	0.091808	0.155328	0.719096	0.150*	
B	0.2121 (17)	0.498 (3)	0.272 (2)	0.064 (7)	
F1	0.259 (3)	0.602 (3)	0.275 (3)	0.287 (18)	
F2	0.2511 (16)	0.3870 (19)	0.2964 (13)	0.180 (9)	
F3	0.1531 (14)	0.523 (3)	0.3305 (18)	0.267 (15)	
F4	0.1865 (18)	0.509 (4)	0.197 (2)	0.278 (16)	

### Atomic displacement parameters (Å^2^)

**Table:**

	*U*^11^	*U*^22^	*U*^33^	*U*^12^	*U*^13^	*U*^23^
Pt	0.0466 (3)	0.0576 (3)	0.0578 (3)	−0.0066 (3)	−0.0007 (10)	0.0042 (9)
CL	0.061 (2)	0.076 (3)	0.128 (5)	−0.019 (2)	0.008 (6)	−0.008 (6)
P1	0.051 (3)	0.067 (3)	0.057 (3)	−0.003 (3)	0.000 (3)	0.006 (3)
P2	0.059 (3)	0.063 (3)	0.060 (3)	0.002 (3)	0.001 (3)	0.018 (3)
C1	0.064 (9)	0.070 (10)	0.087 (11)	−0.005 (9)	0.030 (18)	0.020 (18)
O1	0.090 (9)	0.122 (11)	0.131 (11)	−0.045 (9)	0.004 (19)	−0.057 (19)
C2	0.083 (11)	0.066 (11)	0.055 (13)	0.008 (9)	0.019 (12)	0.030 (12)
C3	0.083 (15)	0.083 (14)	0.078 (14)	0.012 (13)	−0.037 (12)	−0.013 (11)
C4	0.049 (11)	0.080 (12)	0.055 (12)	−0.007 (10)	0.009 (9)	−0.003 (10)
C5	0.091 (13)	0.097 (14)	0.079 (18)	0.032 (11)	−0.012 (16)	0.022 (16)
C6	0.078 (14)	0.059 (12)	0.077 (15)	−0.020 (10)	0.034 (11)	0.001 (10)
C7	0.084 (13)	0.078 (14)	0.060 (12)	0.015 (12)	0.035 (9)	0.025 (12)
C8	0.122 (19)	0.105 (18)	0.072 (14)	0.003 (16)	−0.009 (13)	0.035 (13)
C9	0.16 (2)	0.106 (18)	0.082 (16)	0.038 (17)	−0.057 (16)	−0.005 (13)
C10	0.099 (17)	0.081 (15)	0.105 (17)	0.021 (13)	0.019 (13)	−0.007 (13)
C11	0.102 (19)	0.22 (3)	0.102 (19)	0.05 (2)	−0.014 (16)	0.08 (2)
C12	0.13 (2)	0.077 (16)	0.12 (2)	0.016 (14)	0.062 (17)	0.017 (14)
C13	0.110 (17)	0.12 (2)	0.066 (14)	0.001 (16)	0.002 (12)	0.026 (14)
B	0.061 (13)	0.044 (13)	0.087 (17)	−0.012 (11)	−0.011 (13)	−0.006 (13)
F1	0.37 (4)	0.19 (2)	0.30 (4)	−0.11 (3)	0.10 (3)	−0.08 (2)
F2	0.189 (18)	0.128 (13)	0.22 (2)	0.060 (15)	−0.006 (19)	0.034 (14)
F3	0.18 (2)	0.38 (4)	0.24 (3)	0.07 (2)	0.123 (19)	0.13 (2)
F4	0.25 (3)	0.39 (4)	0.20 (2)	0.14 (3)	−0.08 (2)	−0.04 (2)

### Geometric parameters (Å, º)

**Table:**

Pt—C1	1.813 (18)	C7—C13	1.56 (3)
Pt—CL	2.301 (4)	C7—H7A	0.9700
Pt—P1	2.341 (5)	C7—H7B	0.9700
Pt—P2	2.348 (5)	C8—H8A	0.9600
P1—C2	1.812 (17)	C8—H8B	0.9600
P1—C3	1.804 (18)	C8—H8C	0.9600
P1—C4	1.794 (16)	C9—H9A	0.9600
P2—C5	1.80 (2)	C9—H9B	0.9600
P2—C6	1.808 (18)	C9—H9C	0.9600
P2—C7	1.775 (17)	C10—H10A	0.9600
C1—O1	1.120 (17)	C10—H10B	0.9600
C2—C8	1.50 (3)	C10—H10C	0.9600
C2—H2A	0.9700	C11—H11A	0.9600
C2—H2B	0.9700	C11—H11B	0.9600
C3—C9	1.52 (3)	C11—H11C	0.9600
C3—H3A	0.9700	C12—H12A	0.9600
C3—H3B	0.9700	C12—H12B	0.9600
C4—C10	1.52 (2)	C12—H12C	0.9600
C4—H4A	0.9700	C13—H13A	0.9600
C4—H4B	0.9700	C13—H13B	0.9600
C5—C11	1.46 (4)	C13—H13C	0.9600
C5—H5A	0.9700	B—F1	1.21 (3)
C5—H5B	0.9700	B—F2	1.25 (3)
C6—C12	1.51 (2)	B—F3	1.30 (3)
C6—H6A	0.9700	B—F4	1.20 (3)
C6—H6B	0.9700		
			
C1—Pt—CL	177.0 (12)	C12—C6—H6A	108.5
C1—Pt—P1	92.4 (8)	C12—C6—H6B	108.5
C1—Pt—P2	93.3 (8)	H6A—C6—H6B	107.5
CL—Pt—P1	87.2 (2)	C13—C7—H7A	109.0
CL—Pt—P2	87.1 (2)	C13—C7—H7B	109.0
P1—Pt—P2	174.10 (17)	H7A—C7—H7B	107.8
O1—C1—Pt	178 (3)	C2—C8—H8A	109.5
C2—P1—Pt	111.4 (8)	C2—C8—H8B	109.5
C3—P1—Pt	111.9 (7)	C2—C8—H8C	109.5
C4—P1—Pt	116.7 (6)	H8A—C8—H8B	109.5
C2—P1—C4	106.4 (9)	H8A—C8—H8C	109.5
C2—P1—C3	103.9 (11)	H8B—C8—H8C	109.5
C3—P1—C4	105.6 (10)	C3—C9—H9A	109.5
C5—P2—Pt	111.5 (10)	C3—C9—H9B	109.5
C6—P2—Pt	113.7 (6)	C3—C9—H9C	109.5
C7—P2—Pt	112.0 (7)	H9A—C9—H9B	109.5
C5—P2—C6	108.3 (10)	H9A—C9—H9C	109.5
C5—P2—C7	104.3 (12)	H9B—C9—H9C	109.5
C6—P2—C7	106.4 (10)	C4—C10—H10A	109.5
P1—C2—C8	110.5 (14)	C4—C10—H10B	109.5
P1—C3—C9	114.2 (16)	C4—C10—H10C	109.5
P1—C4—C10	115.7 (14)	H10A—C10—H10B	109.5
P2—C5—C11	115.7 (17)	H10A—C10—H10C	109.5
P2—C6—C12	115.1 (14)	H10B—C10—H10C	109.5
P2—C7—C13	112.8 (14)	C5—C11—H11A	109.5
P1—C2—H2A	109.5	C5—C11—H11B	109.5
P1—C2—H2B	109.5	C5—C11—H11C	109.5
P1—C3—H3A	108.7	H11A—C11—H11B	109.5
P1—C3—H3B	108.7	H11A—C11—H11C	109.5
P1—C4—H4A	108.4	H11B—C11—H11C	109.5
P1—C4—H4B	108.4	C6—C12—H12A	109.5
P2—C5—H5A	108.4	C6—C12—H12B	109.5
P2—C5—H5B	108.4	C6—C12—H12C	109.5
P2—C6—H6A	108.5	H12A—C12—H12B	109.5
P2—C6—H6B	108.5	H12A—C12—H12C	109.5
P2—C7—H7A	109.0	H12B—C12—H12C	109.5
P2—C7—H7B	109.0	C7—C13—H13A	109.5
C8—C2—H2A	109.5	C7—C13—H13B	109.5
C8—C2—H2B	109.5	C7—C13—H13C	109.5
H2A—C2—H2B	108.1	H13A—C13—H13B	109.5
C9—C3—H3A	108.7	H13A—C13—H13C	109.5
C9—C3—H3B	108.7	H13B—C13—H13C	109.5
H3A—C3—H3B	107.6	F4—B—F3	111 (3)
C10—C4—H4A	108.4	F4—B—F2	120 (3)
C10—C4—H4B	108.4	F3—B—F2	108 (3)
H4A—C4—H4B	107.4	F4—B—F1	101 (4)
C11—C5—H5A	108.4	F3—B—F1	107 (3)
C11—C5—H5B	108.4	F2—B—F1	109 (3)
H5A—C5—H5B	107.4		

### Hydrogen-bond geometry (Å, º)

**Table:**

*D*—H···*A*	*D*—H	H···*A*	*D*···*A*	*D*—H···*A*
C5—H5*B*···O1^i^	0.97	2.75	3.45 (2)	129
C13—H13*B*···F2^ii^	0.96	2.43	3.27 (3)	147
C4—H4*A*···F2	0.97	2.56	3.48 (3)	159
C7—H7*A*···F4^iii^	0.97	2.67	3.46 (3)	140
C11—H11*B*···F3	0.96	2.75	3.47 (3)	133
C6—H6*A*···F1^ii^	0.97	2.81	3.67 (4)	147

Symmetry codes: (i) *x*−1/2, −*y*+1, *z*; (ii) −*x*+1/2, *y*, *z*+1/2; (iii) −*x*, −*y*+1, *z*+1/2.
